# Rye sprout as a host for the rapid transient production of a recombinant protein using a geminivirus DNA-containing expression vector

**DOI:** 10.5511/plantbiotechnology.25.0515a

**Published:** 2025-12-25

**Authors:** Sakihito Kitajima, Shigeto Morita, Kohki Natsuhara

**Affiliations:** 1 Department of Applied Biology, Kyoto Institute of Technology, Matsugasaki Sakyo-ku, Kyoto, Kyoto 606-8585, Japan; 2 Graduate School of Life and Environmental Sciences, Kyoto Prefectural University, Kyoto, Kyoto 606-8522, Japan; 3 Biotechnology Research Department, Kyoto Prefectural Agriculture, Forestry and Fisheries Technology Center, Seika, Kyoto 619-0244, Japan

**Keywords:** agroinfiltration, amplicon, rye (*Secale cereale*), sprout, wheat dwarf virus

## Abstract

The agroinfiltration technique using sprouts as a host is one of the most cost-effective, efficient, and rapid methods for producing recombinant proteins. We previously reported that radish sprouts were the best host for this purpose. To find suitable alternative sprouts comparable to radish sprouts, we investigated rye sprouts using a *wheat dwarf virus* (a geminivirus) DNA-containing expression vector. Various rye cultivars were tested, and Raitaro and Ryokuhiyo sprouts exhibited the highest enhanced green fluorescent protein (EGFP) productivity. When agroinfiltrated after a 5-d cultivation period, including 1 day of seed imbibition, approximately 1.8 mg of EGFP was produced per gram fresh weight of leaf in areas exhibiting EGFP fluorescence. This yield is comparable to that of mature leaves from *Nicotiana benthamiana* and radish sprouts. However, only a limited number of leaves produced the protein, and production was confined to areas near the leaf tips. Elevated production levels were observed in the guard cells of stomata and at wounded sites via microneedling, suggesting that the limiting factors for protein production may involve the entry of *Agrobacterium* into the leaves and/or the subsequent transfer of T-DNA into the plant cells.

## Introduction

Mature leaves of *Nicotiana benthamiana* are widely recognized as effective hosts for the transient production of recombinant proteins, primarily due to their high infection and expression efficiencies with the agroinfiltration technique. The use of recently developed expression vectors containing geminivirus genomic DNA fragments significantly enhances protein production by enabling DNA replication following transfer into the plant nucleus via the *Agrobacterium* T-DNA system. This approach can yield up to 3.7 mg of enhanced green fluorescent protein (EGFP) per gram of fresh weight (FW) of leaf (Yamamoto et al. 2018; for other recent studies, see references cited in Kim et al. 2024). A notable constraint of this system is that *N. benthamiana* requires approximately five weeks of growth after germination before agroinfiltration can be initiated. This extended cultivation period requires a substantial growing area and specialized equipment to enable continuous mass production of recombinant proteins.

Using sprouts as a host in a transient production system significantly shortens the cultivation period, making it one of the most cost-effective, efficient, and rapid methods for producing recombinant proteins.

The following criteria should be considered when selecting plant species as hosts for sprouts: they should demonstrate high gene transfer efficiency and protein productivity; there should be a large supply of seeds available year-round at a low cost; germination and subsequent growth should be rapid and synchronous; and the biomass produced per cultivated area should be high.

We previously tested the sprouts of three species from the Fabaceae family, three species from the Brassicaceae family, and six cultivars of radish (*Raphanus sativus*). Our findings indicated that radish sprouts are exceptionally effective hosts for transient protein production (Kitajima et al. 2020). In this system, agroinfiltration is performed 5 days after the seeds are imbibed, and the cotyledons are harvested 3 days later for protein extraction. In total, only 8 days are needed after seed imbibition begins. When using an expression vector developed by Yamamoto et al. (2018), which contains genomic DNA fragments from the bean yellow dwarf virus (BeYDV), a type of geminivirus, this system can yield 0.7 mg of EGFP per g FW cotyledon, resulting in a total of 4.2 mg of EGFP in the growing area of an 8-cm diameter single cultivation cup. This yield is comparable to that of Rubisco protein.

In recombinant protein production, whether using microbial, animal, or plant hosts, the host often produces insufficient amount of the desired protein for various reasons. In such cases, changing the host species is one of the most frequently considered options. Having multiple species to choose from is advantageous, even when using sprouts as a host. We aimed to evaluate Poaceae (monocotyledon) sprouts as alternatives to radish sprouts, offering a host that is both phylogenetically and physiologically distinct. The main part of a Poaceae sprout is a true leaf, while a radish sprout, being a eudicotyledon, has cotyledons. Due to these differences, we expect that the two types of sprouts may yield different protein production results in some cases.

There are reports of transient expression in monocotyledons using T-DNA expression vectors that incorporate the genome of the *wheat dwarf virus* (WDV), a geminivirus that infects Poaceae plants (Gil-Humanes et al. 2017; Tian et al. 2023; Wang et al. 2017) and has been reviewed several times (Bhattacharjee and Hallan 2022; Hefferon 2014; Zaidi and Mansoor 2017). However, these studies were not aimed at mass production of recombinant proteins in sprouts.

To achieve transient mass production of recombinant proteins in sprouts of monocotyledons, we tested nine species of Poaceae in a pilot experiment: rye (*Secale cereale*), Japanese barnyard millet (*Echinochloa esculenta*), wheat (*Triticum aestivum*), proso millet (*Panicum miliaceum*), barley (*Hordeum vulgare*), *Sorghum bicolor*, oat (*Avena sativa*), rice (*Oryza sativa*), and maize (*Zea mays*). These species were selected based on their alignment with the abovementioned criteria, although it remained unknown whether they could produce large amounts of recombinant protein in sprouts. While not as efficient as *N. benthamiana* mature leaves or radish sprouts, rye sprouts demonstrated relatively high protein productivity, followed by sprouts of barley, oats, and maize. In this paper, we present the results of transient protein production achieved through agroinfiltration using rye sprouts as the host, utilizing an expression vector that incorporates fragments of the WDV genome DNA.

## Materials and methods

### Plant materials

Rye cultivars Raitaro, Lahitolle, King, and Ryokuhiyo were purchased from Takii Seed Co., Ltd. (Kyoto, Japan). Cultivars Harumidori and Dash were purchased from Kaneko Seed Co., Ltd. (Gunma, Japan). Cultivar Haruichiban and wheat-rye hybrid Raikokko III (Tuckerbox) were purchased from Snow Brand Seed Co., Ltd. (Hokkaido, Japan).

### Plasmid construction

**Figure 1:**
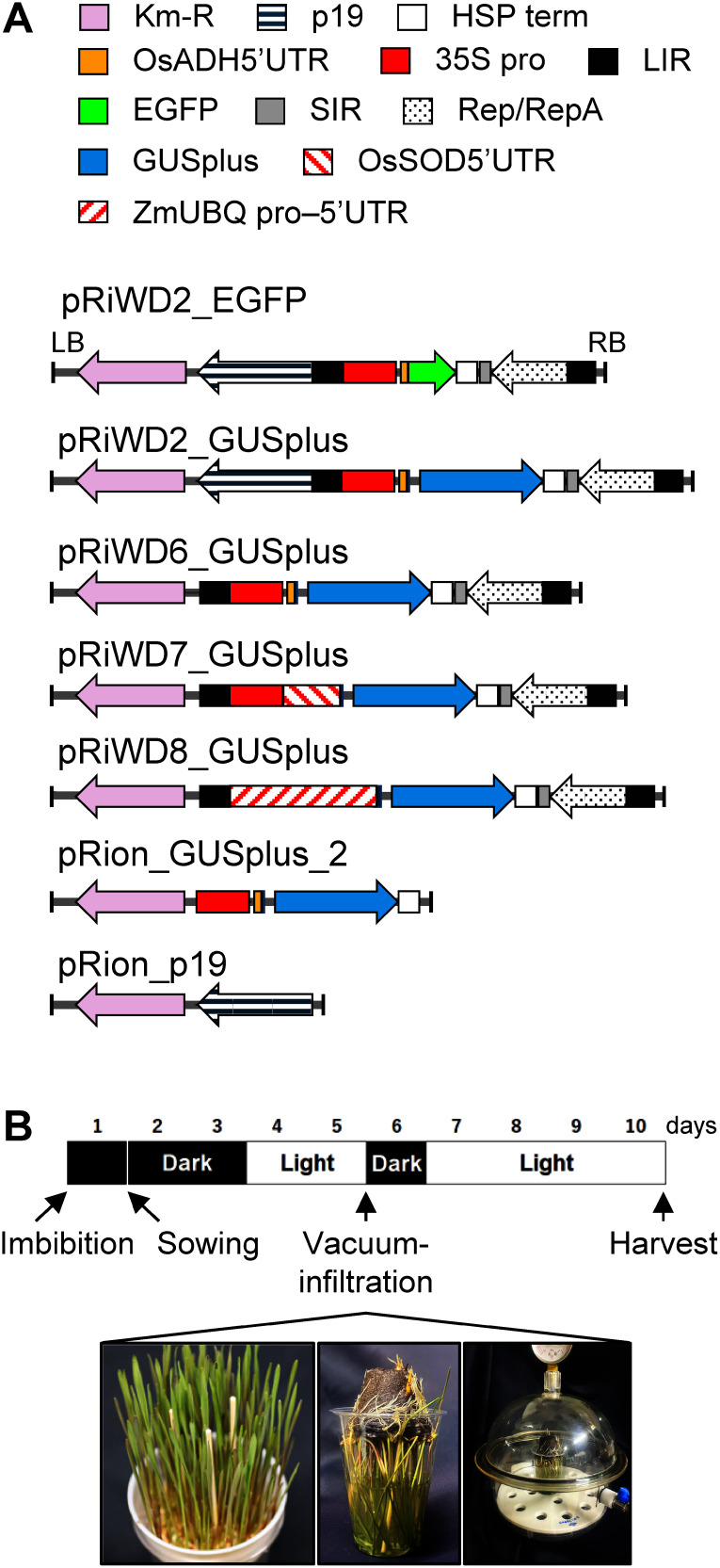
Figure 1. Agroinfiltration procedure for rye sprouts. (A) Expression plasmids used. All plasmids are based on the pRI201 binary vector. The Km-R consists of nopaline synthase (NOS) promoter, *NPTII* gene, and NOS terminator. P19 consists of 35S promoter, *OsADH* 5′ UTR, p19 coding sequence, and HSP terminator. (B) Schedule of agroinfiltration. Photographs are, from the left, sprouts on the day of vacuum infiltration, sprouts dipped in *Agrobacterium* suspension, and their vacuum infiltration in the chamber. The diameter of the cultivation cup is 8 cm.

A DNA fragment coding GUSplus, a variation of β-glucuronidase (GUS) containing an intron to prevent the production of active GUS protein in *Agrobacterium*, was cloned from pCambia-1305 (Abcam, Cambridge, UK). Rice superoxide dismutase (OsSOD) 5′ UTR plus first intron (position 1356–2138, Genbank accession No. L19434) was cloned from p35SINTGUS (Morita et al. 2012). Maize ubiquitin (ZmUBQ) promoter plus 5′ UTR was cloned from pUbiGUSPlus (Addgene #64402). DNA fragments coding p19, rice alcohol dehydrogenase (OsADH) 5′ UTR, and WDV genome (SIR, Rep/RepA, and LIR) were synthesized according to Genbank accession No. NP_062901, Sugio et al. (2008), and Wang et al. (2017), respectively. These DNA fragments were inserted into T-DNA vector pRI201 (Takara-bio, Shiga, Japan) using In-Fusion technology (Takara-bio). The structures of the created plasmids are shown in Figure 1A.

### Rye sprout cultivation

The seeds were imbibed for 1 day in water and put on Jiffy-7 pellets (φ 42 mm, Sakata Seed Co., Yokohama, Japan) that absorbed 2 g l^−1^ of Powder Hyponex (Hyponex Japan Co., Osaka, Japan) solution (Figure 1B). Then, they were cultivated for 2 days in darkness, followed by 2 days of illumination with a white fluorescent lamp (∼1,500 lux) for the purpose of greening. During cultivation, the hydroponic solution was supplied from underneath the pellets. Sprouts were maintained at 25°C during cultivation.

### Rye sprout agroinfiltration

The agroinfiltration of rye sprouts using a vacuum chamber was performed according to the method of Kitajima et al. (2020) for radish sprouts, with some modifications. The *Agrobacterium tumefaciens* strain EHA105, which contains the expression plasmid, was cultured and resuspended in an infiltration buffer [10 mM MgCl_2_, 10 mM MES-KOH (pH 5.6), 0.0025% Silwet L-77, 10 mM sodium ascorbate, 0.25 mM acetosyringone] adjusted to absorbance at 600 nm (Abs_600_) of 0.2. When co-infiltrating two separate *Agrobacterium* strains with different plasmids, a 1 : 1 mixture was prepared, with each strain adjusted to Abs_600_=0.1. For infection of rye sprouts, the leaves were dipped upside down in 20% acetone for 4 min and rinsed with distilled water, dipped in the suspension of *Agrobacterium* carrying expression plasmid(s), and then infiltrated by vacuuming four times for 5 min at approximately –0.1 MPa. Sprouts were further cultivated in darkness for 1 day, followed by illumination for 4 days. The leaves were then harvested for GUS or GFP assay, or protein extraction.

### GUS assay

Histochemical GUS assays were performed according to Sonoda et al. (2009) and de-colored with 70% ethanol for photography.

### EGFP fluorescence

The EGFP fluorescence was captured using a SC52 filter (Fujifilm, Tokyo, Japan) under excitation by a blue LED (472 nm). ImageJ software (Schneider et al. 2012) was used to measure the area exhibiting EGFP fluorescence. Welch’s *t*-test was performed using Excel (Microsoft, Redmond, WA, USA). Adjustment of *p*-values by the Benjamini–Hochberg method (Benjamini and Hochberg 1995) was performed using R version 4.4.2 (https://www.R-project.org/). The significance level of *p*-value was set at 0.05.

### SDS-PAGE and immunoblotting

Leaf pieces were homogenized in extraction buffer [50 mM potassium phosphate and 1 mM EDTA at pH 7.0 supplemented with 0.1% (v/v) β-mercaptoethanol] and soluble and insoluble protein fractions were separated by centrifugation at 18,000×g at 4°C for 15 min. Immunoblotting was performed after SDS-PAGE according to Coligan et al. (1995). Antibodies used were the rabbit anti-GFP polyclonal antibody (product code 598MS; MBL, Nagoya, Japan) and alkaline phosphatase-conjugated goat anti-rabbit antibody (product code ab6722; Abcam, Tokyo, Japan). A mixture of 5-bromo-4-chloro-3-indolyl phosphate and nitro blue tetrazolium (Promega, Madison, WI, USA) was used for color development. For preparation of purified EGFP protein, EGFP was produced in radish sprouts, as described by Kitajima et al. (2020), and the soluble protein fraction of the cotyledon was extracted as described above and loaded onto Q-sepharose XL resin (GE Healthcare, Piscataway, NJ, USA). The EGFP was eluted with the extraction buffer supplemented with 0.1 M KCl. Protein concentration was determined according to Bradford (1976) with bovine serum albumin as the standard. ImageJ software was used to measure band intensity, and linear regression analysis of band intensity was conducted using Excel.

## Results

### WDV-derived DNA fragments enhance recombinant protein production in agroinfiltration of rye sprouts

**Figure 2:**
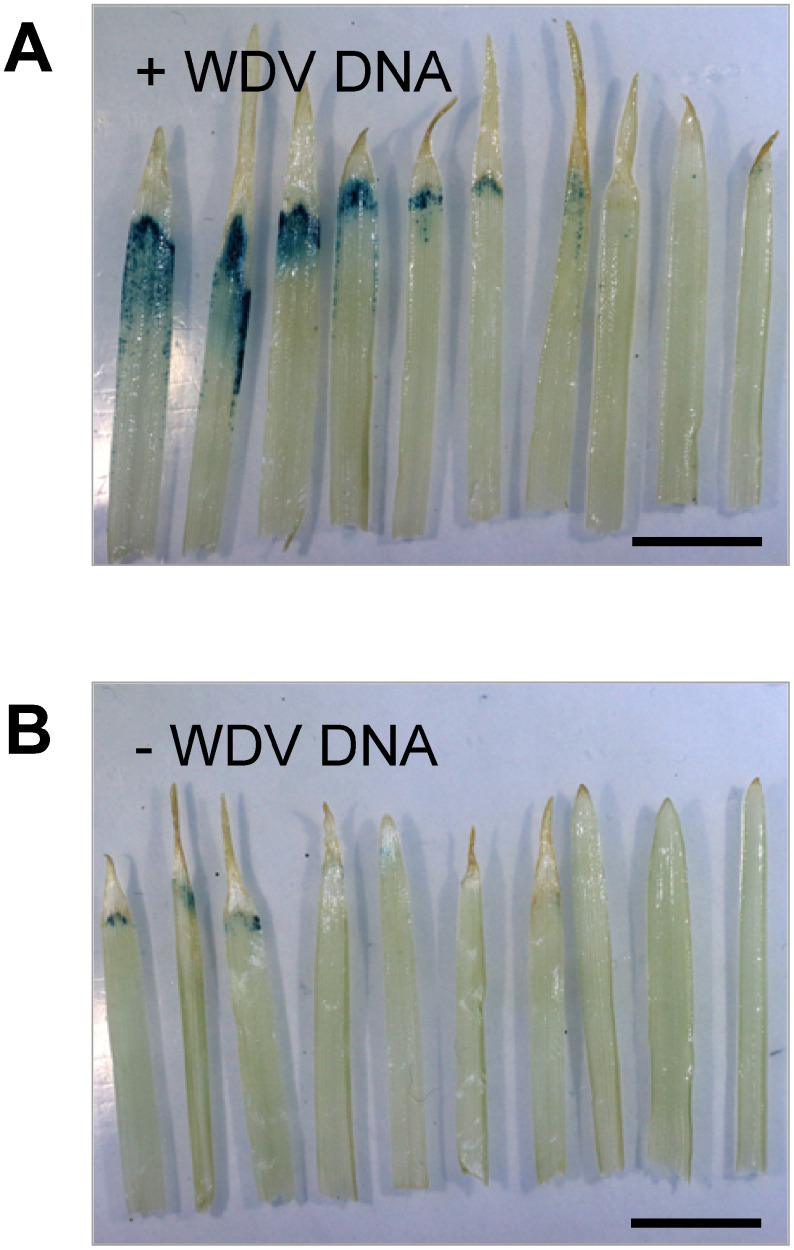
Figure 2. GUS staining of rye sprout leaves infiltrated with plasmids containing or lacking WDV genome fragments. Aerial parts of sprouts were infiltrated with a suspension containing *Agrobacterium* carrying either (A) pRiWD6_GUSplus or (B) pRion_GUSplus_2, along with those carrying pRion_p19. The 10 leaves exhibiting the highest GUS staining are shown from a randomly selected set of 20. The experiments were repeated three times with similar results. The scale bar represents 10 mm.

The schedule for agroinfiltration is illustrated in Figure 1B. We chose the rye cultivar Raitaro for its affordability and easy availability, priced at 2,510 JPN per kg in 2024. At the time of infiltration, the height of the aerial part was approximately 9 cm. To assess whether the inclusion of WDV fragments (SIR, Rep/RepA, and LIR) in the expression vectors improved the production of recombinant protein, we tested two plasmids. The first plasmid, pRiWD6_GUSplus (Figure 1A), contains Cauliflower mosaic virus 35S promoter, *OsADH* 5′ UTR, and GUSplus, along with WDV genome fragments. The *OsADH* 5′ UTR was reported to enhance protein translation in monocotyledon cells (Sugio et al. 2008). The second plasmid, pRion_GUSplus2 (Figure 1A), has the WDV genome fragments removed from pRiWD6_GUSplus. Both plasmids were subjected to agroinfiltration separately. Additionally, a third plasmid, pRion_p19, which produces the p19 protein known to suppress gene silencing (Qiu et al. 2002), was co-infiltrated alongside pRion_GUSplus2 and pRiWD6_GUSplus. Out of the 20 randomly selected leaves, the 10 leaves that exhibited relatively strong GUS staining are shown in Figure 2. The leaves with the introduced pRiWD6_GUSplus construct showed wider staining area, indicating that the WDV genome fragments improved production of the recombinant protein, as expected. However, only about half of the 20 leaves infiltrated with pRiWD6_GUSplus showed staining, and staining intensity varied from intense to faint. In addition, staining was restricted to the area near the leaf tips.

### Impact of the promoter and 5′ UTR on recombinant protein productivity

**Figure 3:**
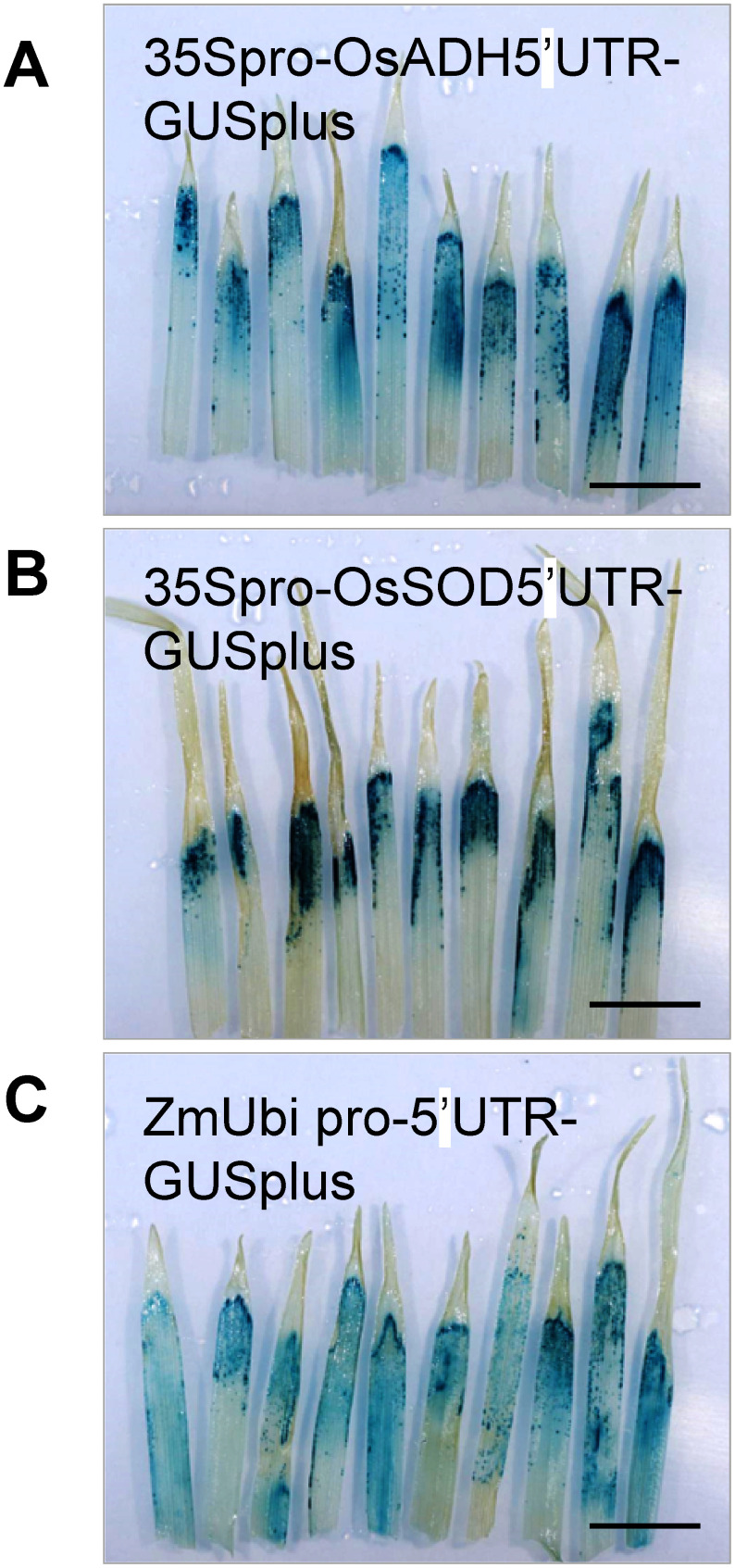
Figure 3. GUS staining of rye sprout leaves infiltrated with three different promoter–5′ UTR constructs. Aerial parts of the sprouts were infiltrated with a suspension containing *Agrobacterium*, carrying three different promoter–5′ UTR-GUS constructs: (A) pRiWD6_GUSplus, (B) pRiWD7_GUSplus, and (C) pRiWD8_GUSplus, along with those carrying pRiWD2_EGFP. The figure shows the 10 leaves with the highest GUS staining, selected from the leaves that exhibited EGFP fluorescence. The experiments were repeated four times with similar results. The scale bar represents 10 mm.

To evaluate the impact of the promoter and 5′ UTR on recombinant protein productivity, we tested two other plasmids. The first plasmid, pRiWD7_GUSplus, contains the 35S promoter along with the *OsSOD* 5′ UTR, which includes its first intron. This UTR has been reported to enhance protein production in monocotyledons (Morita et al. 2012). The second plasmid, pRiWD8_GUSplus, contains the *ZmUBQ* promoter and its 5′ UTR, commonly used for producing recombinant proteins in monocotyledons (Christensen et al. 1992). We co-infiltrated the plasmid pRiWD2_EGFP, which contains the p19 and the 35S promoter – *OsADH* 5′ UTR – EGFP. Leaves exhibiting EGFP fluorescence were selected for GUS staining. The results indicate the stained regions were predominantly located near the leaf tips, similar to observations made with pRiWD6_GUSplus (Figure 3). Therefore, the plasmid with the 35S promoter and *OsADH* 5′ UTR was used in subsequent experiments.

### Impact of rye cultivar on recombinant protein productivity

**Figure 4:**
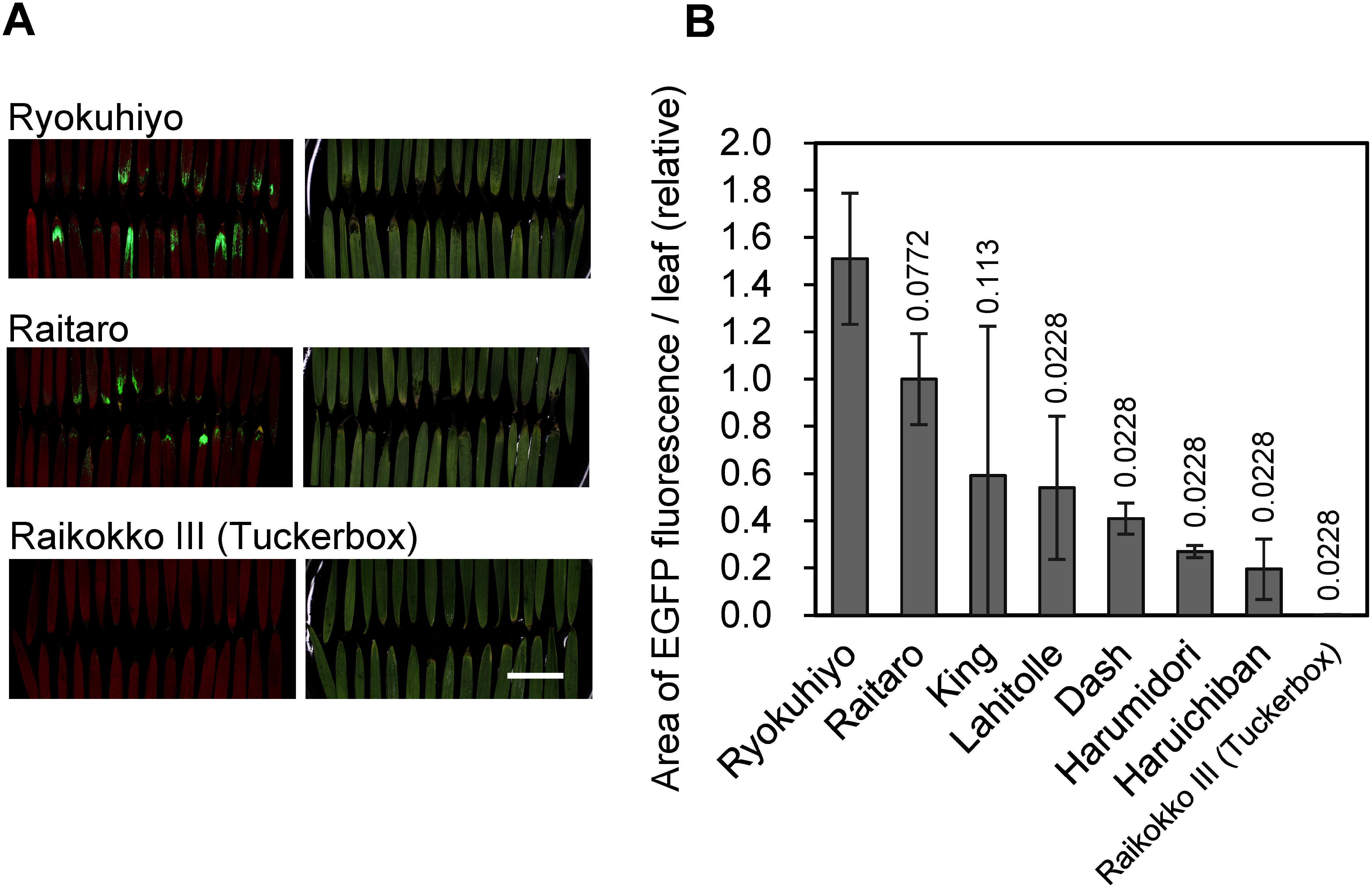
Figure 4. EGFP fluorescence in sprouts of rye cultivars infiltrated with pRiWD2_EGFP. (A) Representative EGFP fluorescence of the two rye cultivars Ryokuhiyo and Raitaro, and a wheat–rye hybrid Raikokko III (Tuckerbox). Left panel, EGFP (green) and chlorophyll (red) fluorescence; right panel, white-light image. The scale bar represents 20 mm. (B) Area of EGFP fluorescence per leaf, with all values normalized to the mean value of Raitaro (2.7 mm^2^ per leaf). The mean values and standard deviations are shown from three replicates, each consisting of approximately 34 randomly selected leaves. *p*-values, obtained from the Welch’s *t*-test for comparisons with Ryokuhiyo and adjusted by the Benjamini–Hochberg method, are presented above the bars.

To evaluate the protein productivity of different rye cultivars, we introduced the pRiWD2_EGFP construct into seven affordable and readily available rye cultivars, including Raitaro, as well as one wheat–rye hybrid. The results showed that the mean area exhibiting EGFP fluorescence was highest in the cultivar Ryokuhiyo, measuring 1.5 times that of Raitaro, which was the second highest; however, the difference between the two cultivars was not significant. Additionally, EGFP fluorescence in Ryokuhiyo was still confined to the region near the leaf tips (Figure 4A, B). In contrast, the mean area exhibiting EGFP fluorescence in the cultivars Lahitolle, Dash, Harumidori, Haruichiban, and the wheat–rye hybrid Raikokko III (Tuckerbox) was significantly lower than that of Ryokuhiyo. Notably, EGFP fluorescence was barely detectable in Raikokko III (Tuckerbox). Based on these results, rye cultivar Raitaro was chosen for further analysis.

### Impact of cultivation period on recombinant protein productivity

**Figure 5:**
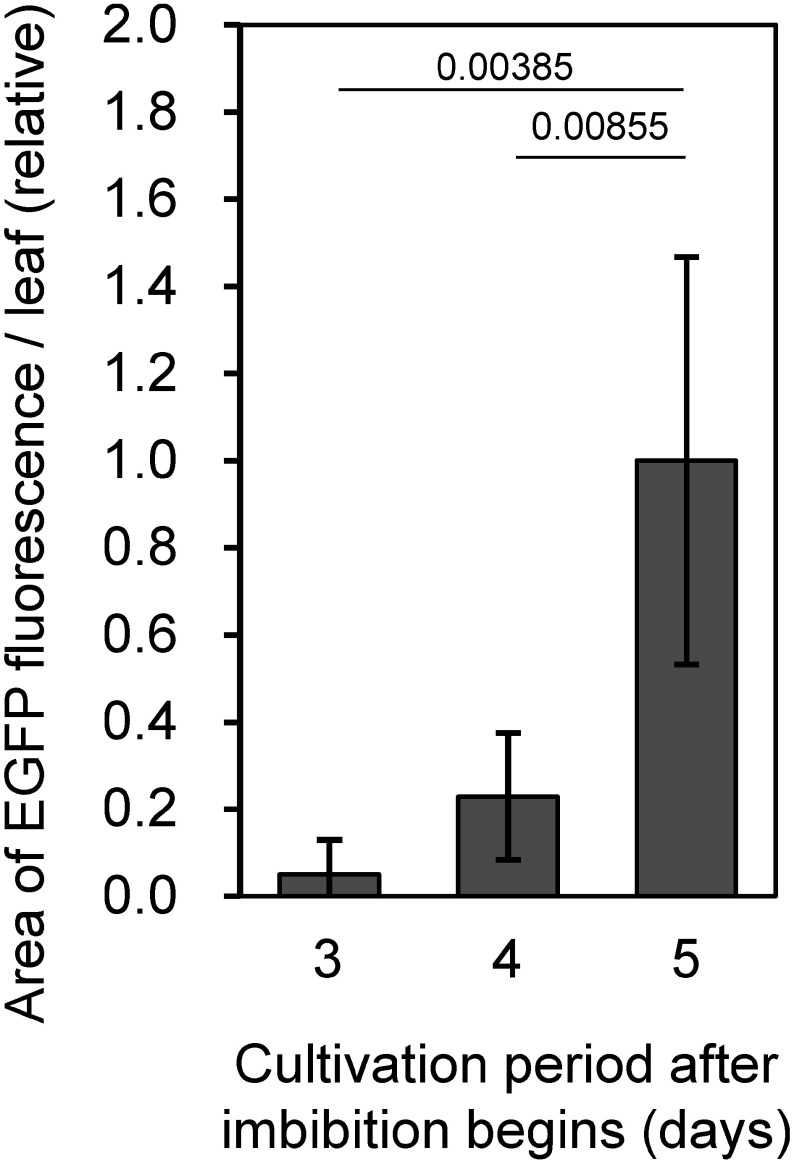
Figure 5. EGFP fluorescence in rye sprouts infiltrated with pRiWD2_EGFP at different cultivation periods. Mean values and standard deviations of the EGFP fluorescence area per leaf are shown, normalized to the mean value from the 5-d cultivation period (0.42 mm^2^ per leaf). Each sample includes six replicates, each consisting of approximately 35 randomly selected leaves. *p*-values from Welch’s *t*-test are presented above the bars.

To assess whether the cultivation period could be shortened, we compared EGFP production after 3 and 4 days of cultivation, with illumination fixed at 2 days, to the standard 5-d cultivation schedule outlined in Figure 1B. The area exhibiting EGFP fluorescence was generally smaller in the samples with the shorter cultivation periods (Figure 5). As a result, we decided to maintain a 5-d cultivation period for subsequent analyses.

### Amount of EGFP produced in rye sprouts

**Figure 6:**
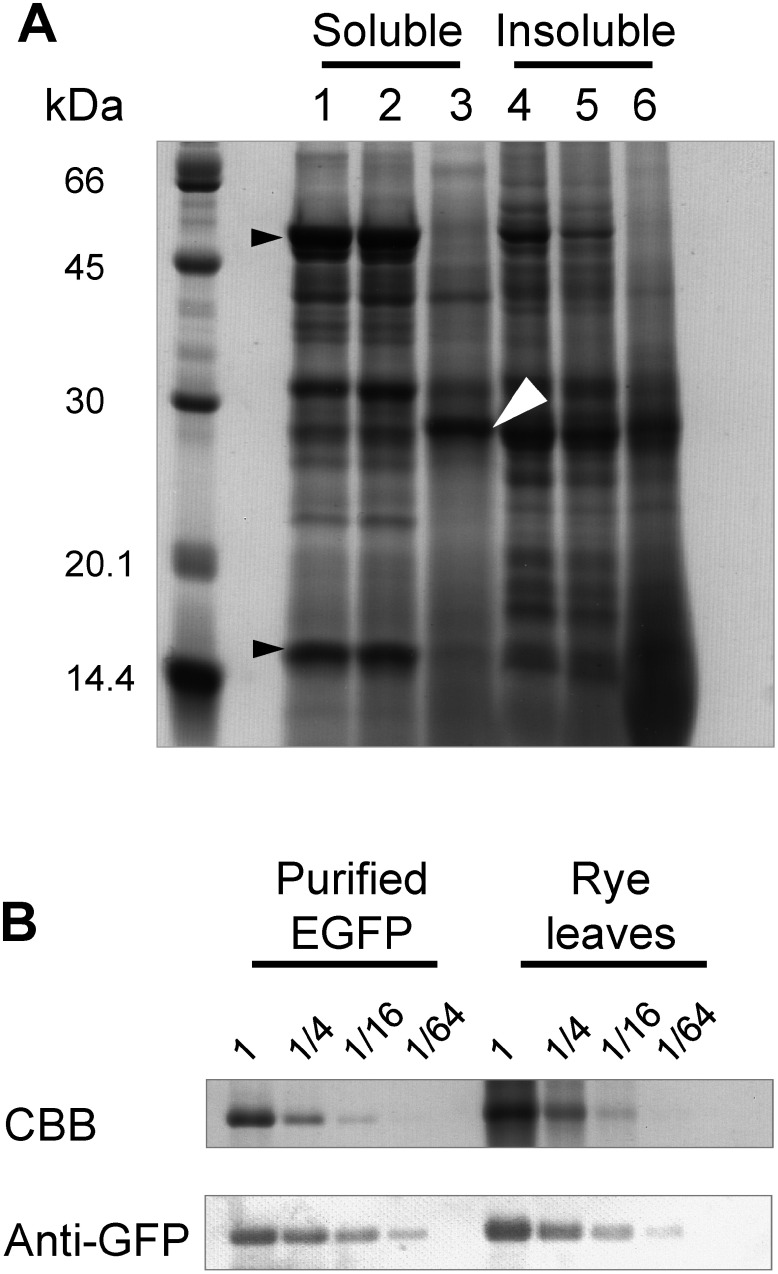
Figure 6. Quantification of EGFP amount at the site exhibiting EGFP fluorescence of rye sprout leaves infiltrated with pRiWD2_EGFP. (A) SDS-PAGE of total soluble (lane 1, 2, 3) and insoluble (4, 5, 6) protein fractions from rye sprout leaves. 1 and 4, non-infiltrated leaves; 2 and 5, infiltrated leaves that did not exhibit EGFP fluorescence; and 3 and 6, infiltrated leaves that exhibited EGFP fluorescence. Total soluble and insoluble protein fractions, obtained from 1 mg of FW leaves, were electrophoresed separately, followed by Coomassie brilliant blue (CBB) staining. White and black arrowheads indicate EGFP and Rubisco subunits, respectively. (B) Detection of EGFP with anti-GFP antibody. For CBB staining, the total soluble protein fraction from 0.48 mg of FW leaves at sites exhibiting EGFP fluorescence, along with its serial dilutions (1/4, 1/16, 1/64), was electrophoresed. As a control, 0.53 µg of purified recombinant EGFP and its serial dilutions (1/4, 1/16, 1/64) were also electrophoresed. For the immunoblotting, one-tenth of the amount of protein used for CBB staining was applied.

To assess the amount of recombinant protein produced at the site of production, sections of leaves infiltrated with pRiWD2_EGFP that exhibited EGFP fluorescence were cut, and the proteins were analyzed using SDS-PAGE (Figure 6A). The soluble protein fraction of both non-infiltrated leaves and leaves that were infiltrated but did not exhibit EGFP fluorescence showed a high accumulation of Rubisco. In contrast, the soluble fraction from the area with EGFP fluorescence showed a significant decrease in Rubisco protein levels. Furthermore, a 27-kDa protein was identified as the predominant protein in this fraction. Immunoblotting with an anti-GFP antibody confirmed that this protein band corresponds to EGFP (Figure 6B). When compared to the band intensity of purified EGFP, the amount of EGFP present in the EGFP fluorescence-positive region of rye was estimated to be 1.8 mg of EGFP per g FW of leaf.

### GUSplus production in guard cells and near microneedled sites

**Figure 7:**
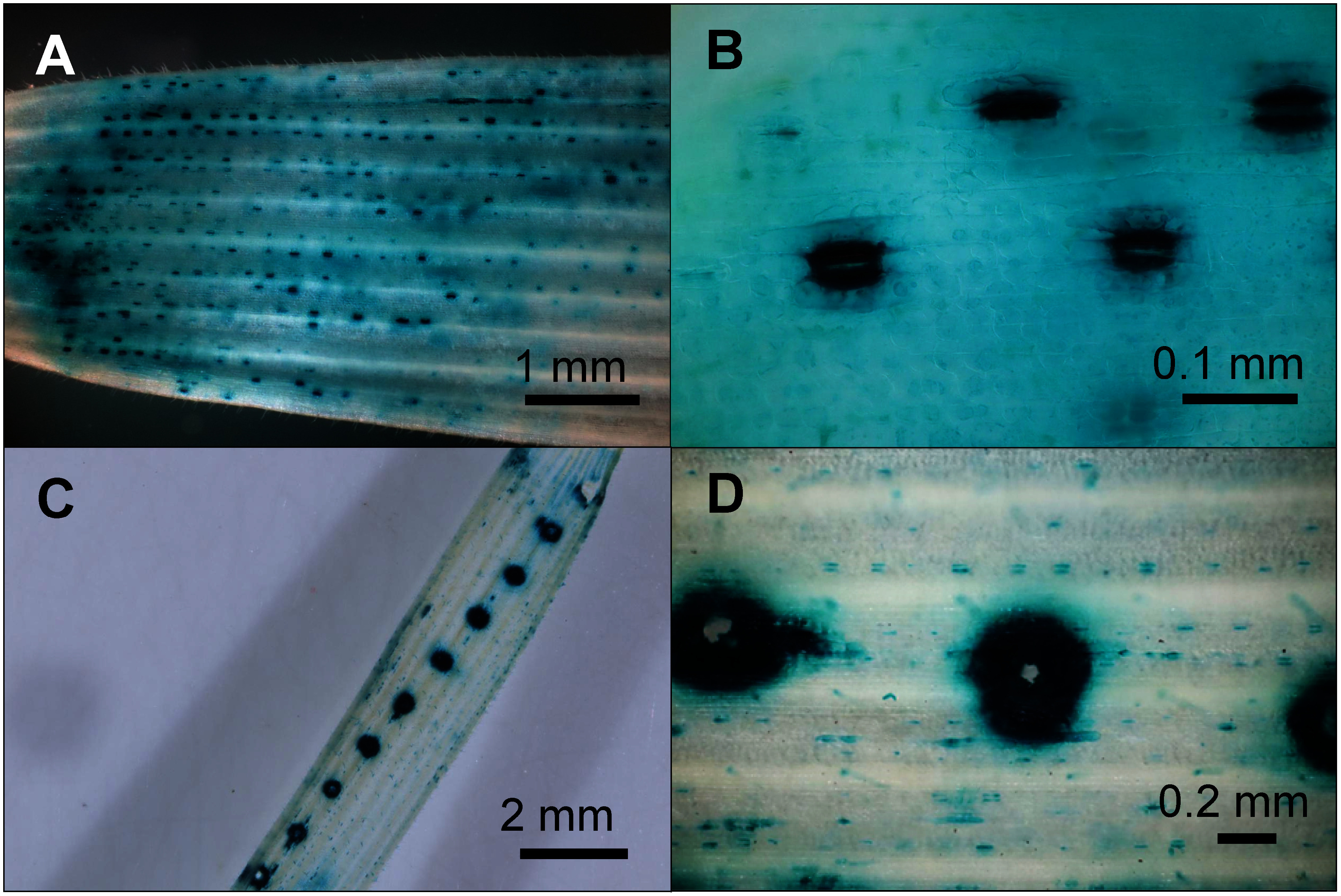
Figure 7. GUS staining of guard cells and wounded areas of rye sprout leaves infiltrated with pRiWD2_GUSplus. Rye sprout leaves (A, B) unwounded and (C, D) wounded with a microneedle roller prior to infiltration.

Microscopic analysis of GUS-stained sites in pRiWD2_GUSplus-infiltrated sprouts revealed a large number of highly stained spots (Figure 7A), which were identified as stomatal guard cells (Figure 7B). This indicates that GUSplus productivity in surrounding cells, such as other epidermal cells and mesophyll cells, which constitute the majority of the leaf, was relatively low compared to guard cells.

Previous study has indicated that EGFP production in citrus leaves increases when the leaves are wounded with a microneedle roller, which creates numerous small wounds, prior to infiltration with *Agrobacterium* containing an EGFP plasmid (Acanda et al. 2021). Similarly, intense GUS staining was observed near the edges of wounded areas in rye sprouts when they were also treated with a microneedle roller before infiltration (Figure 7C, D).

## Discussion

### High-level production of recombinant protein in rye sprouts

In this study, we investigated the use of sprouts of rye, a monocotyledon, to explore alternative plant species for a transient recombinant protein production system that is as high-producing, cost-effective, efficient, and rapid as radish sprouts. When the expression plasmid containing the WDV genome fragments and the 35S promoter-*OsADH* 5′ UTR-EGFP was transiently introduced into rye sprouts, production of EGFP at the EGFP fluorescence-positive sites was approximately 1.8 mg per g FW leaf (Figure 6). This result is comparable to EGFP production in *N. benthamiana* mature leaves, which yielded 3.7 (Yamamoto et al. 2018), 1.5 (Lee et al. 2024), and 0.7–1.2 (Kim et al. 2024) mg per g FW leaf, as well as in radish sprouts, which yielded 0.7 mg per g FW cotyledon (Kitajima et al. 2020).

### Protein production limited to a small region of the sprout

Unfortunately, however, recombinant protein production was restricted to a small number of leaves, despite the sprouts in a culture pot exhibiting similar morphological characteristics (Figure 1B). Furthermore, production was confined to small areas near the tips of those leaves (Figures 2–4). The host sprout cultivar influenced protein productivity; however, even with the two most productive cultivars, Raitaro and Ryokuhiyo, the issue of heterogeneous production sites remained unaddressed (Figure 4).

### Possible factors suppressing protein production in rye sprouts

*Agrobacterium* is generally recognized as less effective at infecting Poaceae plants. This is thought to be due to their epidermal cuticular wax, elevated silica content, reduced intercellular space volume (Andrieu et al. 2012; Kaur et al. 2021), and production of antimicrobial benzoxazinoid compounds (Bakera et al. 2020; Sahi et al. 1990; Zhang et al. 2000).

In leaf regions with GUS staining, guard cells exhibited more intense staining compared to surrounding cells (Figure 7A, B). This is possibly due to the thinness of the guard cell walls, which must deform to open and close the stomata. The wounding of leaves caused by microneedle rollers resulted in significant GUS staining at their edges (Figure 7C, D), possibly through enhanced entry of *Agrobacterium* into the leaves. These results suggest that one limiting factor in our system is *Agrobacterium* entry into the leaves and/or T-DNA transfer into cells, rather than transcription and translation in the plant cells. Additionally, these results indicate that some pre-treatment might enhance protein production throughout the entire leaf of the sprouts in the future.

### Possible pre- or post-treatment of rye sprout to expand the protein-producing region

Using microneedling on all leaves for industrial-scale protein production would be difficult. Therefore, one potential approach is to investigate the effects of adding chemicals that inhibit the biosynthesis of cell wall components, such as lignin and cellulose, as well as the biosynthesis of the epidermal cuticle. Some of these compounds are available commercially as pesticides. For example, triaziflam (Idetop™) (Grossmann et al. 2001) and indaziflam (Alion™) (Brabham et al. 2014) are herbicides that inhibit cellulose biosynthesis. Because these herbicides are relatively inexpensive, pre-treating sprouts with them would not significantly raise the costs of protein production.

In addition, although they do not use geminivirus DNA-containing vectors, several methods have been reported to enhance transient protein production in Poaceae plant sprouts. For instance, Burman et al. (2020) recommended lowering the growing temperature to 20°C for rice sprouts. Xu et al. (2023) found that incubating barley sprouts in high humidity for 1 day and then in darkness for 2 days after agroinfiltration significantly improved GUS production.

### Alternative ways: Suppressing the immune response in rye sprout

Our observation that Rubisco protein was significantly decreased at the sites of EGFP production (Figure 6A) suggests that the cells were forced to modify their intracellular physiology. This may arise as an immune response to *Agrobacterium* infection or from the production of excessive exogenous protein. Engineering immune-related reactions may thus affect the region of protein production. Recent research has identified several techniques to enhance the efficiency of *Agrobacterium* infection in *N. benthamiana* and other plants by suppressing the host’s immune response. These methods include treating host plants with tenoxicam, an anti-inflammatory drug for human (Choi et al. 2022), and treating with the pathogenic bacterium *Xanthomonas citri* ssp. *citri*, specifically for citrus plants (Jia and Wang 2014a, b). Additionally, treatment with the AvrPto protein, an effector protein from the pathogen *Pseudomonas syringae*, has also been explored (Das et al. 2020).

Engineered strains of *Agrobacterium* that suppress the host’s immune response may also affect the region of protein production. These include the *Agrobacterium* strain carrying the genes for ACC deaminase (*acdS*) and GABA transaminase (*gabT*), which produce enzymes that degrade ACC (the ethylene precursor) and GABA, respectively (Nonaka et al. 2019). Additionally, effector proteins from the pathogenic *Pseudomonas syringae*, such as AvrPto, AvrPtoB, or HopAO1, can be introduced into plant cells from *Agrobacterium* via an engineered type III secretion system (for wheat, alfalfa, and switchgrass; Raman et al. 2022). Furthermore, a mutation in *Agrobacterium* EF-Tu protein, which is part of the pathogen-associated molecular pattern, can suppress pattern-triggered immunity in host plants (Yang et al. 2023).

### Alternative ways: Increasing the infection activity of *Agrobacterium*

In addition, techniques are reported which increase the infection activity of *Agrobacterium*: by constitutively-active mutation in *Agrobacterium* virG protein, which is a response regulator of signaling pathway through detecting acetosyringone to induced *vir* genes’ expression (this mutation alone or in combination of expression of *nahG*, which encodes salicylate hydroxylase) (Jeong et al. 2024; Pazour et al. 1992; Scheeren-Groot et al. 1994); and mutation in *Agrobacterium* repA protein, which regulates copy number of expression plasmids (Szarzanowicz et al. 2025). These techniques may help expand the protein production area in rye sprouts.

### Different EGFP productivity among the cultivars

Finally, we discuss how the productivity of EGFP varied among the different cultivars (Figure 4). The differences may be attributed to the intracellular physiology or anatomical structure of the leaves, but we do not yet have a clear explanation. Notably, EGFP fluorescence was barely detectable in the wheat–rye hybrid Raikokko III (Tuckerbox). This low productivity may be linked to genes inherited from the wheat genome. Identifying these genetic factors would be valuable for improving agroinfiltration in rye and other Poaceae plants in the future.

In conclusion, we utilized rye sprouts as a host to develop a cost-effective, efficient, and rapid system for transient protein production, serving as an alternative to radish sprouts. The expression plasmid containing the WDV genome proved effective, yielding approximately 1.8 mg of EGFP per g FW leaf when the sprouts were used for protein production 5 days after seed imbibition. Despite the fact that Poaceae are generally considered less susceptible to *Agrobacterium* infection, this productivity is comparable to that of mature leaves of *N. benthamiana* and radish sprouts, although production occurred in a small, limited region of the sprout. Our next objective will be to facilitate protein production across the entire leaf region.

## Abbreviations

EGFP: enhanced green fluorescent protein
FW: fresh weight
GUS: β-glucuronidase
OsADH: *Oryza sativa* alcohol dehydrogenase
OsSOD: *Oryza sativa* superoxide dismutase
UTR: untranslated region
WDV: wheat dwarf virus
ZmUBQ: *Zea mays* ubiquitin
